# [^18^F]AlF-PSMA-11 PET in diagnosing prostate cancer: a head-to-head comparison with [^68^Ga]Ga-PSMA-11 PET and an exploration of dual-phase scanning

**DOI:** 10.1186/s41824-024-00217-5

**Published:** 2024-09-09

**Authors:** Xiao Li, Mingming Yu, Jian Yang, Danni Li, Rou Li, Juanli Mao, Changjing Zuo, Zeying Liang, Qiang Li, Chao Cheng

**Affiliations:** 1 Department of Nuclear Medicine, Shanghai Changhai Hospital, Shanghai, 204333 China; 2 Shanghai Institute of Applied Physics, Chinese Academy of Sciences, Shanghai, 201800 China; 3 Department of Radiology, The Affiliated People’s Hospital of Ningbo University, Ningbo, 315040 Zhejiang China; 4 School of Medicine, Shanghai University, Shanghai, 200444 China

**Keywords:** [^18^F]AlF-PSMA-11, [^68^Ga]Ga-PSMA-11, PSMA PET, Dual-phase scans, Prostate Cancer

## Abstract

**Purpose:**

To evaluate the physiological distribution and tumour detection ability of [^18^F]AlF-PSMA-11 positron emission tomography (PET) dual-phase scans in patients with prostate cancer (PCa).

**Methods:**

As a retrospective study, clinical and PET data of PCa patients who underwent dual-phase [^18^F]AlF-PSMA-11 PET of routine scan (45–50 min) and delayed scan (120 min) from November 2020 to June 2021 were collected, and physiological and pathological regions of interest were quantified to determine the time-dependent maximum standardized uptake value (SUV_max_) of [^18^F]AlF-PSMA-11. Part of the above subjects who underwent [^68^Ga]Ga-PSMA-11 PET in the following 6 months were included in a head-to-head comparison. The difference with a p-value < 0.05 was defined as statistical significance. Diagnosis accuracy of primary and metastatic lesions was measured referring to the surgical findings, pathology, and follow-up imaging.

**Results:**

[^68^Ga]Ga-PSMA-11 and [^18^F]AlF-PSMA-11 were of the comparable uptake in glands in head, but the latter was of a significant lower distribution in liver and spleen. For the 25 patients initially diagnosed with prostate cancer and 3 patients with biochemical recurrence after radical surgery, the SUV_max_ of the primary lesions, lacrimal glands, parotid glands and submandibular glands was higher at 120 min compared to that at 45–50 min, but not a significant difference. SUV_max_ of the liver, spleen and bladder decreased significantly at 120 min, but the bladder SUV_max_ remained higher than that of primary lesions. SUV_max_ of the kidneys and centrum was the same in dual-phase scans. For the 31 primary lesions detected in [^18^F]AlF-PSMA-11 PET, both the SUV_max_ of the two phases kept the positive correlation with PSA, Gleason score and initial risk stratification. For the 39 distant metastatic lesions, 94.87% accuracy of routine scan and 100% accuracy of delayed scan were acquired, and 7.14% patients (2/28) benefited from the dual-phase [^18^F]AlF-PSMA-11 scans that revealed novel information on metastatic lesions compared to the routine scan.

**Conclusion:**

[^18^F]AlF-PSMA-11 PET expanded the time window and further decreased metabolic background of [^68^Ga]Ga-PSMA-11 PET. The dual-phase scan of [^18^F]AlF-PSMA-11 PET can benefit prostate cancer diagnosis via providing more PSMA-specific information.

## Introduction

Prostate cancer (PCa) is one of the most common malignancies in men worldwide and the second leading cause of cancer-related death in males (Siegel et al. 2023). Accurate staging and localization is essential for selecting appropriate treatment strategies, such as active surveillance, radical prostatectomy, radiation therapy, hormone therapy and watchful waiting (Mottet et al. 2021). Currently recommended imaging staging methods for PCa include magnetic resonance imaging (MRI), computed tomography (CT) and bone scintigraphy. MRI is mainly used for evaluating the extent of primary tumour infiltration and pelvic lymph node metastasis due to its excellent soft tissue contrast (Williams et al. 2022). CT and bone scintigraphy are used to assess distant metastasis, but they have low sensitivity and may result in missed diagnoses (Maurer et al. 2016; Zhao and Ji 2022). As a whole-body functional imaging modality, positron emission tomography (PET) has been widely used in the staging of malignant tumours. In the majority of PCa cases, prostate-specific membrane antigen (PSMA) is an overexpressed cell surface transmembrane protein. Since PSMA exhibits high affinity with PSMA-targeting molecular probes, PSMA ligand-targeted PET imaging has significant advantages in the diagnosis, staging, follow-up and prognostic evaluation of PCa (Hofman et al. 2020; Koerber et al. 2017).

Various PSMA-targeted PET imaging agents have been developed, including [^68^Ga]Ga-PSMA-11 (Koerber et al. 2017; Uprimny et al. 2017), [^18^F]F-PSMA-1007 (Kuten et al. 2020; Rahbar et al. 2018) and [^18^F]F-DCFPyL (Morris et al. 2021; Rowe et al. 2022). Among them, [^68^Ga]Ga-PSMA-11 is widely accepted in clinical practice due to its convenient preparation and availability of the radionuclide (Koerber et al. 2017). However, the shorter half-life and larger positron emission energy of Ga-68 compared to F-18 may affect diagnostic accuracy (Dietlein et al. 2015). [^18^F]AlF-labeled PSMA-HBED-CC (hereafter, [^18^F]AlF-PSMA-11), a novel PSMA-targeted PET imaging agent and an alternative to [^68^Ga]Ga-PSMA-11, replaces [^68^Ga]Ga with [^18^F]AlF because the radionuclide F-18 has a more suitable half-life for PET applications without altering the framework of the PSMA-HBED-CC precursor. Although [^18^F]AlF-PSMA-11 has been deemed an attractive alternative to [^68^Ga]Ga-PSMA-11 for PCa imaging, its advantages and clinical applications have been rarely studied (De Man et al. 2022; van Leeuwen and Emmett 2022). This study aims to understand the tracer metabolism and physiological distribution of [^18^F]AlF-PSMA-11 via a head-to-head comparison with [^68^Ga]Ga-PSMA-11 PET, as well as to investigate the application of [^18^F]AlF-PSMA-11 PET in diagnosing PCa, especially the added value of dual-phase imaging in PCa management.

## Materials and methods

### Study design and patient population

A retrospective analysis of PCa patients who underwent [^18^F]AlF-PSMA-11 PET imaging at Changhai hospital from November 2020 to June 2021 was conducted. The study was approved by the ethics committee of Changhai hospital (Approval No. CHEC2019-090). The data are anonymous and retrospective, so the requirement for informed consent was waived. Inclusion criteria were as follows: (a) histologically or surgically confirmed PCa; (b) availability of Gleason score and PSA results within 2 weeks. For the above patients with a [^68^Ga]Ga-PSMA-11 PET performed within 6 months, the clinical data were further included in the head-to-head comparison with [^18^F]AlF-PSMA-11 PET.

### Imaging protocol

[^18^F]AlF-PSMA-11 was prepared in-house according to our previously reported optimised protocol with a labeling kit of Al-PSMA-11 (Kersemans et al. 2018; Li et al. 2022). Patients received intravenous injection of [^18^F]AlF-PSMA-11 at a dose of 1.85 MBq/kg. After injection, the patients were instructed to drink water and rest quietly for 45–50 min, followed by voiding and lying supine on the PET/CT scanning bed. This study defined imaging at 45–50 min as routine imaging, and imaging at 120 min as delayed imaging. Imaging acquisition was performed using a Siemens Biograph 64 PET/CT scanner. Patients maintained calm breathing, and the scanning range extended from the head to the mid-thigh. A whole-body CT scan was performed first, with a current of 170 mA, a voltage of 120 kV and a slice thickness of 3 mm. Subsequently, PET scanning was performed to acquire 5–6 bed positions, including 1–2 abdominal positions and 1 head position, with a duration of 3 min per bed position. PET images were corrected for CT attenuation and reconstructed using the TrueD system on a post-processing workstation to generate PET/CT fusion images in transverse, coronal and sagittal planes and maximum intensity projection (MIP).

Patients drank water and rested quietly, and all but seven patients were then scanned with a PET/CT again at 120 min. For better soft tissue contrast, the other seven patients underwent a delayed scan at 120 min with an integrated PET/MR scanner (Biograph mMR, Siemens Healthcare) that combines PET and 3.0-T MRI scanners. After a fast and simple MRI scout imaging sequence, a whole-body PET scan (3 min/bed position) was conducted for 5–6 bed positions. MRI was concurrently conducted using the following protocol: T1-weighted 3D volumetric interpolated breath-hold examination with Dixon fat saturation (3D, transversal, TR 4.07 ms, TE 1.28 ms, flip angle 12°, 72 slices, slice thickness 3 mm, field of view 400 × 400, voxel size 1.3 × 1.3 × 3.0 mm^3^), and T2W-BLADE (transversal, TR 3000 ms, TE 89 ms, flip angle 90°, 33 slices, slice thickness 6 mm, field of view 400 × 400, voxel size 1.3 × 1.3 × 6.0 mm^3^). PET data were reconstructed using high-definition PET (3 iterations, 21 subsets; matrix 172 × 172, voxel size 2.3 × 2.3 × 5.0 mm^3^). The Dixon sequence was used to derive MRI-based attenuation correction.

[^68^Ga]Ga-PSMA-11 PET/CT was performed following the same procedure with the routine scan of [^18^F]AlF-PSMA-11 PET/CT.

### Image analysis and interpretation

All images were independently reviewed by two nuclear medicine physicians with more than 8 years of diagnostic experience. The maximum standardised uptake value (SUV_max_) within the region of interest (ROI) was independently delineated on two sets of PET images. Conversion factors for SUV_max_ between PET/CT and PET/MR were calculated using a water phantom in advance of quantitative investigation, and the results showed that the PET counts were equivalent between the two modalities. For the precise analysis, our previous report on the two scanners has confirmed the consistency of quantification (Zhang et al. 2022). All subjects were followed up until December 2023, and the diagnosis accuracy of primary and metastatic lesions was measured referred to the surgical findings, pathology, and follow-up imaging examinations.

### Statistical analysis

The data were analyzed using IBM SPSS 21.0 software. Normally distributed continuous variables were presented as mean ± standard deviation (SD). Graphs were charted with Origin 2024. Independent samples t-test was used for comparing quantitative data. A p-value < 0.05 was considered to indicate statistical significance.

## Results

**Fig. 1:**
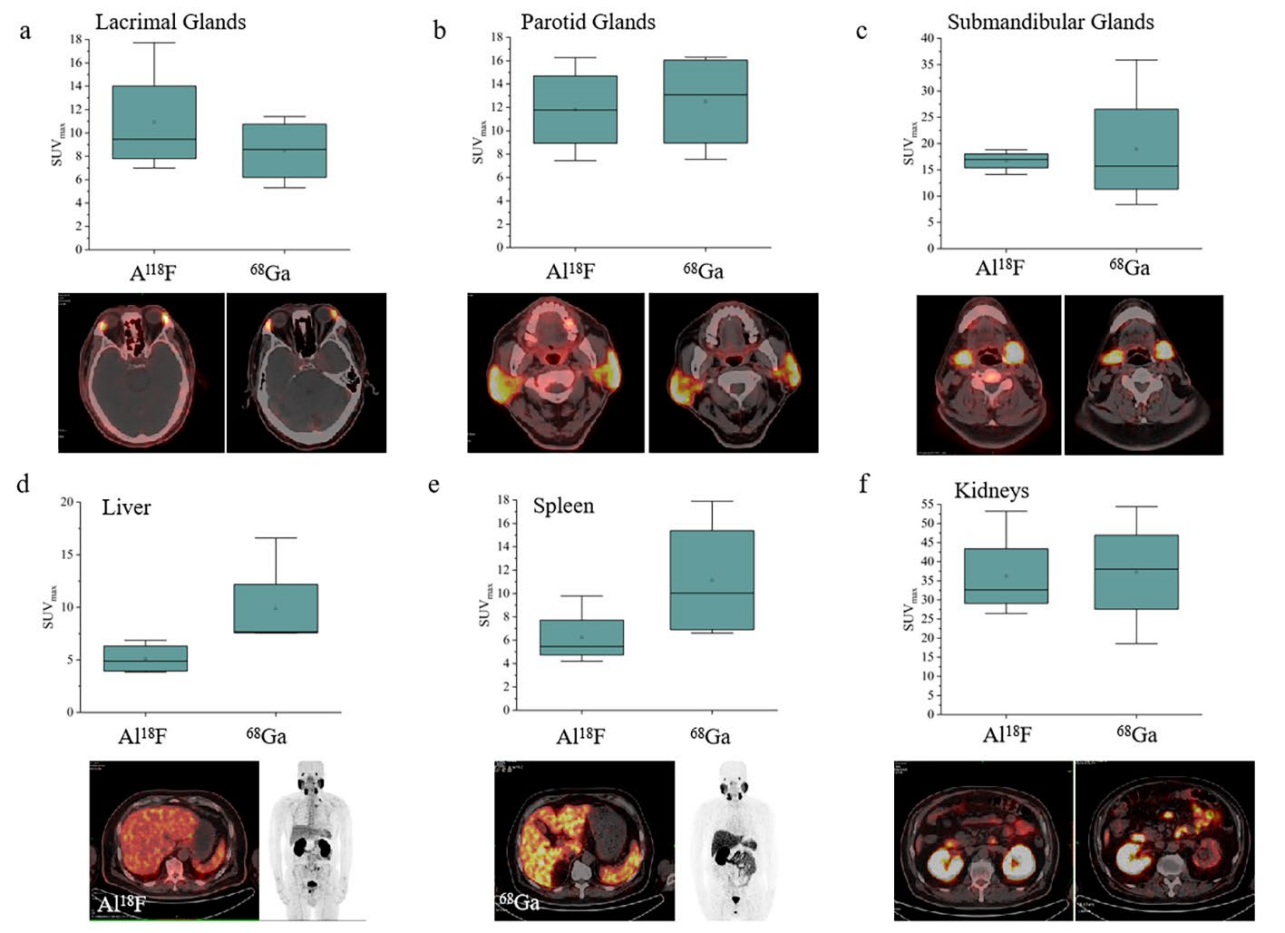
Comparison of tracer uptake between [^18^F]AlF-PSMA-11 PET and [^68^Ga]Ga-PSMA-11 PET in glands in head (**a**, **b** and **c**) and metabolic organs (**d**, **e** and **f**). The representative [^18^F]AlF-PSMA-11 PET images were of a 65-year-old man with prostate cancer, GS 4 + 5 and serum PSA value 45.19 ng/mL, and the [^68^Ga]Ga-PSMA-11 PET was acquired at 4 months later

A total of 28 PCa patients were retrospectively recruited, including 25 patients with initial PCa diagnosis and 3 patients with postoperative biochemical recurrence. Detailed clinical characteristics and pathological results are summarized in Table 1. Among them, 4 patients received a follow-up [^68^Ga]Ga-PSMA-11 PET/CT within 6 months. For the head-to-head comparison of [^18^F]AlF-PSMA-11 PET and [^68^Ga]Ga-PSMA-11 PET, there were comparable tracer uptakes in those glands in head (Fig. 1a-c), however, a significant lower tracer uptake was observed in [^18^F]AlF-PSMA-11 PET of liver and spleen (Fig. 1d-e), when compared with [^68^Ga]Ga-PSMA-11 PET. The confounding factors, such as specific activity and radiochemical purity of PSMA-11-derived radiopharmaceuticals, were strictly controlled, therefore, pharmacokinetics of [^18^F]AlF-PSMA-11 were the only factor in determining the clearer background of metabolic organs in PET imaging. Kidneys were of a similar SUV_max_ in the two scans (Fig. 1f).

**Table 1:** Clinical and pathological characteristics of PCa patients who underwent [^18^F]AlF-PSMA-11 PET

Characteristic and Pathology	Value or Number (Percentage, %)
Age (mean ± SD)	(67.7 ± 7.1) y
PSA (ng/mL)	
≤ 10	11 (39.3)
> 10	17 (60.7)
Gleason score	
≤ 7	13 (46.4)
> 7	15 (53.6)
Distant metastasis	
M0	19 (67.9)
M1	9 (32.1)
Initial risk stratification	
Intermediate risk	11 (39.3)
High risk	17 (60.7)

Table 2 presents the quantitative data of tissues or organs with obvious tracer uptake. In the primary lesions of PCa patients, a comparable quantification was acquired at 45–50 min and 120 min. For the lacrimal glands, parotid glands and submandibular glands, there was a rising trend in SUV_max_ at 120 min compared to that at 45–50 min, but the difference was not significant, pointing to the PSMA-specific [^18^F]AlF-PSMA-11 uptake by these tissues and the extended time window of PSMA PET. Conversely, the liver, spleen and bladder showed a significant decrease in SUV_max_ at 120 min compared to that at 45–50 min, further strengthening the advantage of [^18^F]AlF-PSMA-11 PET with a clearer metabolic background. The diagnosis of pelvic lesions will potentially benefit from the noticeable decrease of bladder SUV_max_ in the delayed imaging even though the value of SUV_max_ remains higher than that of the lesions. Due to the continuous clearance of [^18^F]AlF-PSMA-11, the SUV_max_ of the kidneys was nearly identical in both scans. Overall, the replacement of [^68^Ga]Ga with [^18^F]AlF enabled delayed PSMA-11 PET, leading to an increased tracer uptake or maintained PSMA-specific tracer uptake.

**Table 2:** Quantification of whole-body distribution of [^18^F]AlF-PSMA-11

Location	SUV_max_		t	*P*
	Routine Scan	Delayed Scan		
PCa*	13.54 ± 9.09	13.96 ± 11.56	-0.141	0.888
Lacrimal gland	7.72 ± 3.57	9.69 ± 4.73	-1.651	0.105
Parotid gland	11.70 ± 5.21	13.22 ± 6.99	-0.873	0.387
Submandibular gland	11.62 ± 4.62	12.54 ± 6.44	-0.582	0.564
Liver	4.20 ± 1.42	2.96 ± 1.04	3.336	**0.002**
Spleen	5.56 ± 2.55	3.76 ± 1.60	2.782	**0.004**
Bladder	35.33 ± 24.32	18.10 ± 13.44	3.128	**0.003**
Kidney	31.57 ± 12.21	31.54 ± 16.42	0.006	0.995
Bone (Centrum-L5)	3.43 ± 1.26	3.70 ± 1.78	-0.637	0.527

*SUV_max_ of primary lesions was measured in 25 non-surgical patients, and in cases with multiple primary lesions, the average value of the lesions was obtained

**Fig. 2:**
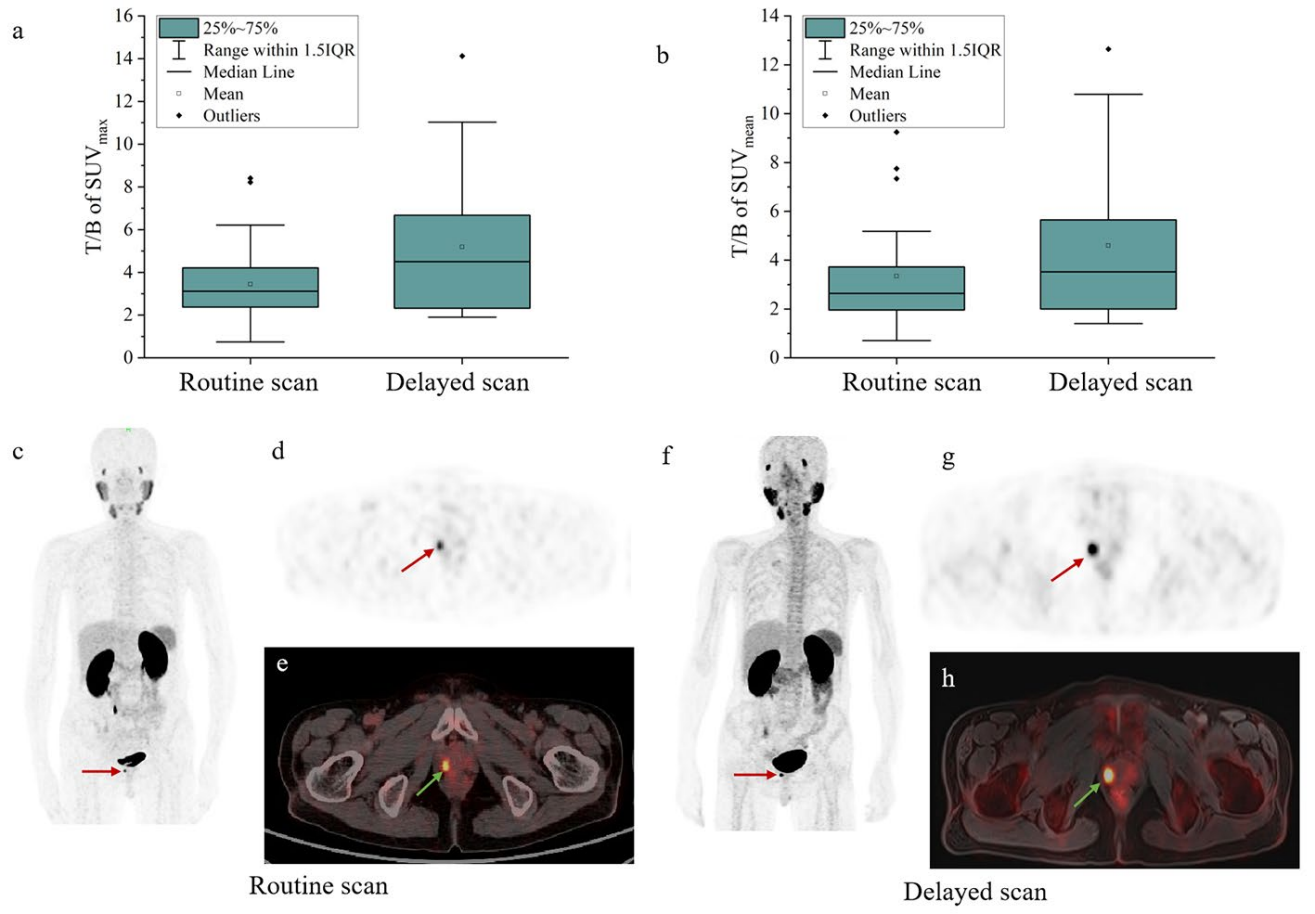
The target to background ratios of SUV_max_ **(a)** and SUV_mean_ **(b)** of [^18^F]AlF-PSMA-11 PET of primary lesions, and images of a 63-year-old man with prostate cancer, GS 3 + 4 and serum PSA value 10.15 ng/mL. In routine imaging, performed 50 min after [^18^F]AlF-PSMA-11 injection, **(c)** PET MIP, **(d)** PET and **(e)** fused PET/CT show increased focal tracer uptake (SUV_max_, 11.40) in the prostatic region (red and green arrows). In delayed imaging, performed 120 min after [^18^F]AlF-PSMA-11 injection, **(f)** PET MIP, **(g)** PET and **(h)** fused PET/MR show increased focal tracer uptake (SUV_max_, 10.61) in the prostatic region (red and green arrows) and expanded tracer uptake range

In evaluating the 25 non-surgically treated patients with [^18^F]AlF-PSMA-11 PET, 31 primary lesions in the prostate were detected by routine imaging at 45–50 min and then confirmed by delayed imaging at 120 min, accordingly, the ratio of target (primary lesion) to background (liver) increased as shown in Fig. 2a (SUV_max_) and Fig. 2b (SUV_mean_). Unlike the unaltered number of lesions, the range of primary lesions tended to expand in the delayed scanning (Fig. 2c-h), standing for a more precise mapping of PSMA-related lesions. Table 3 summarizes the difference in [^18^F]AlF-PSMA-11 uptake between groups divided by PSA, Gleason score or risk stratification. There were comparable values of differential diagnosis by SUV_max_ at 45–50 min and 120 min. For both the quantification at routine and delayed scans, SUV_max_ of primary PCa lesions showed a positive correlation with PSA level, Gleason score and risk stratification at both 45–50 min and 120 min, implying that PSA level, Gleason score and risk stratification continuously served as the inherent factors in determining SUV_max_ of primary lesions in the dual-phase scans of [^18^F]AlF-PSMA-11 PET.

**Table 3:** Correlation of SUV_max_ of primary prostate lesions with PSA, Gleason score and Risk Assessment

Characteristic and Pathology	SUV_max_ at 45–50 min	t	*P*	SUV_max_ at 120 min	t	*P*
PSA		-2.353	0.030		-2.419	0.029
≤ 10	9.74 ± 5.06			11.52 ± 5.31		
> 10	16.44 ± 7.29			20.11 ± 7.70		
Gleason		-2.417	0.029		-2.171	0.049
≤ 7	9.46 ± 4.98			9.50 ± 4.56		
> 7	17.55 ± 8.20			17.54 ± 8.80		
Initial risk stratification		-2.614	0.021		-2.916	0.012
Intermediate	10.17 ± 4.92			10.32 ± 4.13		
High risk	18.94 ± 7.56			19.03 ± 8.35		

Among the 28 cases, a total of 39 distant metastatic lesions were detected in the dual-phase [^18^F]AlF-PSMA-11 scan and then verified with the surgical findings, pathology, and follow-up imaging, such as MRI and [^99m^Tc]Tc-MDP SPECT/CT. For the 39 distant metastatic lesions, 94.87% accuracy of routine scan and 100% accuracy of delayed scan were acquired, and 7.14% patients (2/28) benefited from the dual-phase [^18^F]AlF-PSMA-11 scans that revealed novel information on metastatic lesions compared to the routine scan.

**Fig. 3:**
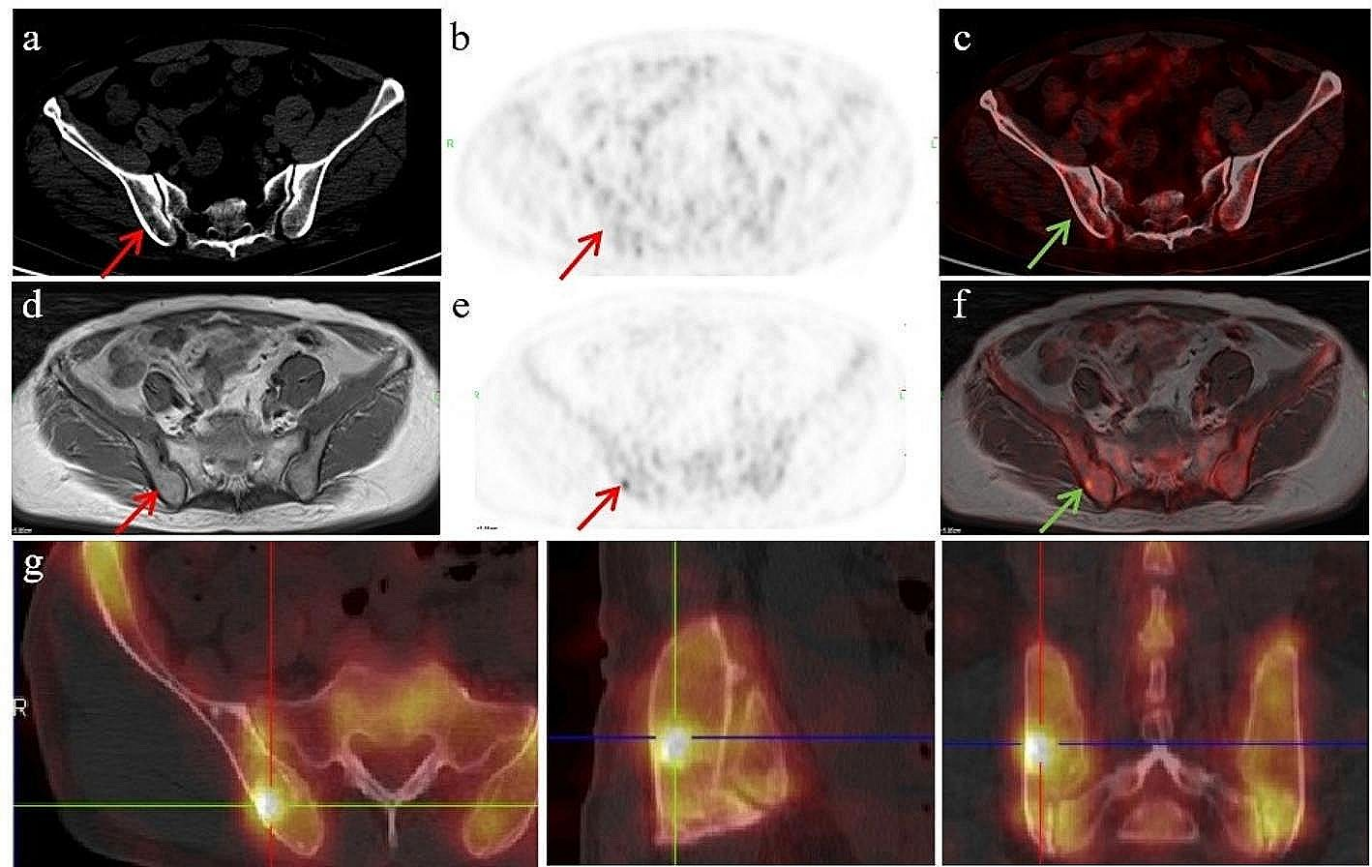
A 56-year-old man with prostate cancer who underwent radical prostatectomy 6 months ago with a serum PSA value of 1.14 ng/mL. The patient did not receive radiation therapy or hormonal therapy after surgery. In routine imaging, performed 50 min after [^18^F]AlF-PSMA-11 injection, **(a)** PET/CT axial CT image shows mild increased bone density on the right iliac bone (red arrow), and **(b)** PET and **(c)** fused PET/CT images show no focal tracer uptake (red and green arrows) in the corresponding area. In delayed imaging, performed 120 min after [^18^F]AlF-PSMA-11 injection, **(d)** PET/MR-T1WI image shows mild restricted signal on the right iliac bone (red arrow), and **(e)** PET and **(f)** fused PET/MR images show tracer uptake (SUV_max_, 5.94) on the right iliac bone (red and green arrows). **(g)** shows the high uptake of ^99m^Tc-MDP in the same focol location in a follow-up SPECT/CT scan performed at 15 months later

**Fig. 4:**
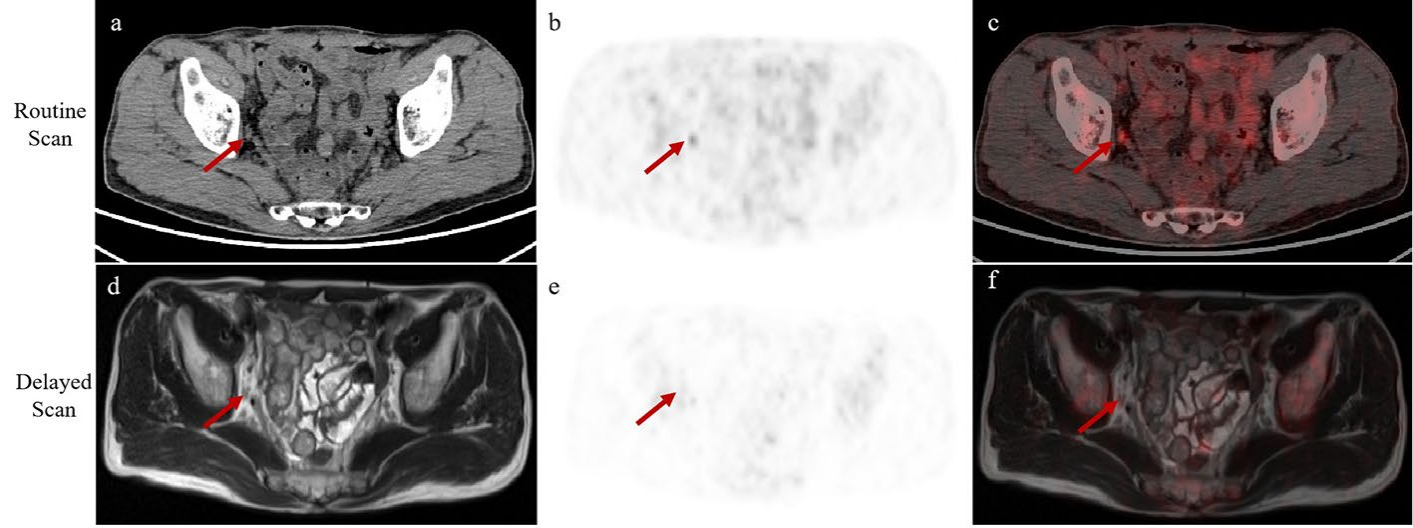
A 63-year-old man with prostate cancer, GS 4 + 4 and serum PSA value of 11.68 ng/mL. In routine imaging, performed 50 min after [^18^F]AlF-PSMA-11 injection, **(a)** axial PET/CT, **(b)** PET and **(c)** fused PET/CT images show tracer uptake (SUV_max_, 5.3) on the right pelvic wall (red arrows). In delayed imaging, performed 120 min after [^18^F]AlF-PSMA-11 injection, **(d)** PET/MR-T1WI, **(e)** PET and **(f)** fused PET/MR images show no tracer uptake in the lymph nodes (red arrows). The final surgical pathology confirms the absence of lymph node metastasis in this patient

Specifically, in a case with a negative early scan (Fig. 3a-c), a solitary bone metastasis (SUV_max_, 5.94) was only revealed by the delayed imaging (Fig. 3d-f). In a follow-up [^99m^Tc]Tc-MDP SPECT/CT scan that was performed at 15 months later, a metastasis at the same anatomy was identified (Fig. 3g). Additionally, one patient exhibited unclear findings, since pelvic lymph node involvement was observed in routine imaging, but reduced tracer uptake to a background level was observed in delayed imaging, indicating non-metastatic lymph nodes (Fig. 4). Lymph node dissection during subsequent surgery confirmed the absence of lymph node metastasis in this patient. Overall, the dual-phase imaging with [^18^F]AlF-PSMA-11 PET give the nuclear medicine physicians more confidence in making a diagnosis on the basis of PSMA correlation.

## Discussion

Due to its pathological over-expression in PCa patients, PSMA has become an ideal target for the diagnosis and treatment of PCa (Farolfi et al. 2021; Lütje et al. 2015). [^18^F]AlF-PSMA-11, a novel PSMA imaging agent, has the same molecular precursor as [^68^Ga]Ga-PSMA-11 but a longer half-life and lower electron emission energy due to the replacement of the radioactive isotope with F-18, resulting in a higher PSMA-positive lesion detection rate compared to [^68^Ga]Ga-PSMA-11 (De Man et al. 2022). In this study, the physiological distribution, tumour detection capability and delayed imaging of [^18^F]AlF-PSMA-11 in PCa patients were demonstrated through dual-phase PET imaging. Although both [^68^Ga]Ga-PSMA-11 and [^18^F]AlF-PSMA-11 were PSMA-11-derived radiopharmaceuticals, the chemical formula of [^18^F]AlF-PSMA-11 was more complex, furthermore, the difference of electric charge of [^18^F][AlF]^2+^ and [^68^Ga]Ga^3+^ potentially affected the pharmacokinetics of tracers. Referring to the head-to-head comparison, PSMA-specificity was not influenced, while replacing [^68^Ga]Ga^3+^ with [^18^F][AlF]^2+^ minimized the metabolic burden of liver and spleen.

Good and stable target uptake is an important prerequisite for the application of targeted molecular probes. The results of this study showed good imaging of [^18^F]AlF-PSMA-11 in primary prostate lesions, lacrimal glands and salivary glands, with high uptake still observed in the delayed imaging performed at 120 min, indicating a favourable and stable biological distribution of [^18^F]AlF-PSMA-11 in target organs. Notably, the SUV_max_ of the liver and spleen significantly decreased in the delayed imaging, which is advantageous for the detection of liver metastases. In contrast, another F-18 labelled PSMA imaging agent, [^18^F]F-PSMA-1007, may not be suitable for the detection of liver metastases due to its hepatic clearance (Rahbar et al. 2018). Maintaining the metabolic characteristics of [^68^Ga]Ga-PSMA-11, [^18^F]AlF-PSMA-11 is also excreted through the kidneys, resulting in high tracer accumulation in the bladder during delayed imaging. Afshar et al. demonstrated that by prolonging the scan time to 3 h and administering a diuretic to reduce the bladder tracer concentration, more occult lesions around the bladder could be detected in delayed [^68^Ga]Ga-PSMA-11 imaging (Afshar-Oromieh et al. 2017). However, further research is needed to determine whether an increased detection rate of lesions around the bladder can be achieved by adopting a longer delay in imaging (e.g., 4 h) without administering a diuretic for patients who have contraindications to diuretics. The longer half-life of F-18 makes it more suitable for PET imaging, allowing for higher resolution even with delayed scans.

The results of this study showed that delayed imaging with [^18^F]AlF-PSMA-11 can clarify the nature of lesions. In one case of biochemical recurrence after radical surgery (Fig. 3), a solitary bone metastasis was detected in the delayed imaging, and careful observation revealed slight bone abnormalities on CT and MR, which was manifested as a bone metastasis in a follow-up MDP SPECT/CT. In another case, a patient suspected of having lymph node metastasis showed significantly reduced uptake in the delayed imaging (Fig. 4), and subsequent surgery confirmed the absence of pelvic lymph node metastasis. Similar results have been reported in previous studies (Afshar-Oromieh et al. 2016, 2017; Rahbar et al. 2018; Sahlmann et al. 2016), where both the SUV_max_ values and target-to-background ratios of malignant lesions significantly increased with time. Such appearance may result from the ability of PSMA receptors binding to the imaging agent is directly proportional to time, whereas the imaging agent in non-target tissues is rapidly cleared over time, thereby improving the target-to-background ratio through delayed imaging. These additive PSMA-specific information resulted from the dual-phase [^18^F]AlF-PSMA-11 PET was hopeful to decrease the necessity of confirmatory examination, such as bone scintigraphy. Although only two cases (7.14%) in this study had changes in tumour staging based on delayed imaging, a larger sample size may reveal more missed lesions with routine imaging compared to delayed imaging with [^18^F]AlF-PSMA-11. Therefore, we recommend extending the scan time appropriately for [^18^F]AlF-PSMA-11 PET, although determining the optimal scan time still requires further expansion of sample studies.

Most literature suggests that the SUV_max_ of PSMA PET is positively correlated with PSA, Gleason score and risk stratification (Ergül et al. 2018; Uprimny et al. 2017). [^18^F]AlF-PSMA-11 maintained the PSMA-specificity of PSMA-HBED-CC, that is to say, the SUV_max_ of primary prostate lesions increased as the PSA, Gleason score and risk level increased, consistent with the literature. In the delayed imaging of [^18^F]AlF-PSMA-11 PET, we found a small number of cases exhibited diffused bone uptake, potentially resulting from F-18 being prone to deposition in the bones, especially in benign conditions such as osteogenesis, joint degeneration and old fractures. Similarly, due to de-labelling or relatively low radiochemical purity, Rauscher et al. found that [^18^F]F-PSMA-1007 detected more benign lesions than [^68^Ga]Ga-PSMA-11 (Rauscher et al. 2020). This requires diagnostic physicians to have extensive clinical experience and make comprehensive judgments based on the patient’s medical history, PSA and Gleason score.

Here, we preliminarily reported the additional value of [^18^F]AlF-PSMA-11, but there are certain limitations to this study. First, since this was a small-sample study, further studies with larger sample sizes are needed to validate the findings, especially the best choice of delayed scans protocol. Second, although our diagnostic physicians have extensive diagnostic experience, there could have been some false positives due to the lack of pathological confirmation for some bone metastatic lesions. Further follow-up or pathological confirmation is needed. Finally, a head-to-head comparison between [^68^Ga]Ga-PSMA-11 and [^18^F]AlF-PSMA-11 was performed on three tumour-free subjects already (Li et al. 2022), and an extensive head-to-head validation performed on four PCa patients was carried out in this study, but a further comparison focused on tumour was more meaningful in exploring the clinical advantages of [^18^F]AlF-PSMA-11 PET.

## Conclusion

This clinical study demonstrated that [^18^F]AlF-PSMA-11 can bind to receptors stably and persistently, meantime, a clearer metabolic background was manifested when compared with [^68^Ga]Ga-PSMA-11 PET, indicating that [^18^F]AlF-PSMA-11 has great potential as an alternative PSMA imaging agent to [^68^Ga]Ga-PSMA-11 since it allows for further characterization of lesions through delayed imaging.

## Data Availability

Data are available from the corresponding author on reasonable request.
